# A preliminary identification of Rf*-A619, a novel restorer gene for CMS-C in maize (*Zea mays* L.)

**DOI:** 10.7717/peerj.2719

**Published:** 2016-11-22

**Authors:** Liu Yongming, Zhao Zhuofan, Lu Yanli, Li Chuan, Wang Jing, Dong Boxiao, Liang Bing, Qiu Tao, Zeng Wenbing, Cao Moju

**Affiliations:** Maize Research Institute, Sichuan Agricultural University, Chengdu, P.R. China

**Keywords:** Cytoplasmic male sterility, Restorer gene, Fertility restoration, Gene targeted marker, Maize

## Abstract

C-type cytoplasmic male sterility (CMS-C) is widely utilized for hybrid maize seed production. However, genetic mechanisms underlying the fertility restoration are very complicated. At present, there is a divergence on the number of fertility restorer genes in maize inbred line A619 for CMS-C. To further elucidate the restoring mechanism of A619, we used genetic analysis and molecular markers to confirm the restorer genes of maize inbred line A619 for C-type male sterile line C48-2 in this study. Firstly, the fertility segregations of (C48-2 × A619)F_2_ populations were investigated under three environments during 2013–2015. The segregation ratio of fertile and sterile plants in the F_2_ population fit to 15:1 via chi-square test and this result suggested that there are two dominant restorer genes in A619 for CMS-C, i.e., *Rf4* and a novel gene named Rf*-A619. Next, based on the sequence differences between *Rf4* and its recessive allelic *rf4*, a novel dominant marker F2/R2 was developed and validated to genotyping *Rf4* in the F_2_ population. Through genotypic analysis, we found that there were a certain amount of fertile individuals without *Rf4* which accounted for 3/16 in the F_2_ population via chi-square test at the 0.05 level. These results provided another proof to sustain that the inbred line A619 contains one additional restorer gene for CMS-C fertility restoration except *Rf4*. At last, we used one SSR marker which is tightly linked with the dominant restorer gene *Rf5* to analyze those fertile plants without *Rf4* in the F_2_ population. The PCR amplification results showed that Rf*-A619 is not allelic to *Rf5* but a novel restorer gene for CMS-C. These results not only provide a basis for the mapping and characterization of a novel restorer gene but also give a new insight into the mechanism of CMS-C fertility restoration.

## Introduction

CMS is a popular phenomenon in plant and fertility restorer genes (*Rf*) and can rescue the fertility of CMS lines, and CMS/*Rf* systems in crops have been successfully utilized for human being because of the heterosis (Hu et al., 2014). To date, many restorer genes in various species have been identified and characterized (Bohra et al., 2016), and this information greatly improves our knowledge of the genetic basis of male sterility and fertility restoration and accelerates the utilization of plant male sterility in practice.

According to the pattern of fertility restoration in CMS hybrid F_1_ progeny, maize cytoplasmic male sterility can be divided into three major types: T (Texas), S (USDA), and C (Charrua) (Beckett, 1971). CMS-T is almost eliminated in seed production due to its vulnerability to the fungus *Helminthosporium maydis* race T (Duvick, 1973). CMS-S male sterility is unstable compared to the other two cytoplasms, and this deficiency limits its application in agriculture (Weider et al., 2009). By contrast, CMS-C owns a stable male sterility and has a positive effect on grain yield (Weider et al., 2009; Stevanovic et al., 2016). As a result, CMS-C is now widely used for seed production. Nevertheless, there is a lack of a firm understanding of the major and minor restorer factors that overcome the deleterious mitochondrial open reading frames in CMS-C. Previous studies have demonstrated that fertility restoration of CMS-C is controlled by two dominant genes, *Rf4* and *Rf5*, which separately located on chromosomes 8 and 5 (Chen, Luo & Ji, 1979; Sisco, 1991; Tang et al., 2001). In addition, *Rf6* and some quantitative trait loci (QTLs) involved in the partial restoration of male fertility for CMS-C have also been identified (Qin, Xu & Dun, 1990; Kohls et al., 2011). Furthermore, as an inhibitor of the *Rf5* restorer gene, ‘*Rf-I*’ has been mapped to chromosome 7 in the sterile line CMS-C77, but it could not prevent *Rf4* function in fertility restoration (Hu et al., 2006). Thus, compared to *Rf5*, *Rf4* can restore fertility of CMS-C lines that have the *Rf-I* inhibitor. *Rf4* is a basic helix-loop-helix transcription factor (Ren et al., 2012). Despite cloning and genetic complementation experiments indicating that GRMZM2G021276 is a candidate gene for CMS-C fertility restoration (Ren et al., 2012), the mechanism by which a transcription factor targeting the nucleus can overcome the deleterious effects of a mitochondrial defect remains mysterious. On the other hand, as a restorer line containing *Rf4*, the inbred line A619 was widely used in the CMS-C fertility restoration studies (Sisco, 1991; Huang et al., 1997; Tang et al., 2001; Ren et al., 2012). However, the number of restorer genes in A619 and their loci have remained controversial. Some studies suggested that *Rf4* is the only restorer gene in A619 and located on chromosome 8 (Tang et al., 2001; Ren et al., 2012). By contrast, Huang et al. (1997) found that the dominant restorer gene in A619 might be located on chromosome 7 and inferred that the inbred line A619 might own another dominant restore gene besides *Rf4*. Moreover, Sisco (1991) not only mapped the restorer gene *Rf4* to chromosome 8 but also inferred that there was one duplicate gene of *Rf4* on chromosome 3 in A619. The above research results hinted A619 rescues the male sterility of CMS-C with highly complicate mechanisms.

In the present study, using genetic analyses and molecular markers, we proved the inbred line A619 possesses two fertility restorer genes, *Rf4* and the novel restorer gene Rf*-A619. Especially, when combined with earlier studies, we found that the function of Rf*-A619 might be affected by the genetic backgrounds of CMS-C lines. These results not only facilitate the mapping of a novel restorer gene but also contribute to the understanding of the mechanism underlying fertility restoration of CMS-C.

## Materials & Methods

### Plant materials

Maize CMS-C male sterile lines C48-2 was used as parent pollinated with inbred line A619 pollen. The hybrid of (C48-2 × A619)F_1_ was totally fertility restored, then self-pollinated to obtain its F_2_. The F_2_ populations were cultivated in the winter of 2013–2014 at Xishuangbanna (21°95′N latitude, 100°76′E longitude) and at Chengdu (29°98′N latitude, 102°99′E longitude) in the spring of 2015. In this experiment, the CMS-C line C48-2 exhibited complete male sterility and the inbred line A619 had normal male fertility.

### Phenotyping male fertility

**Figure 1:**
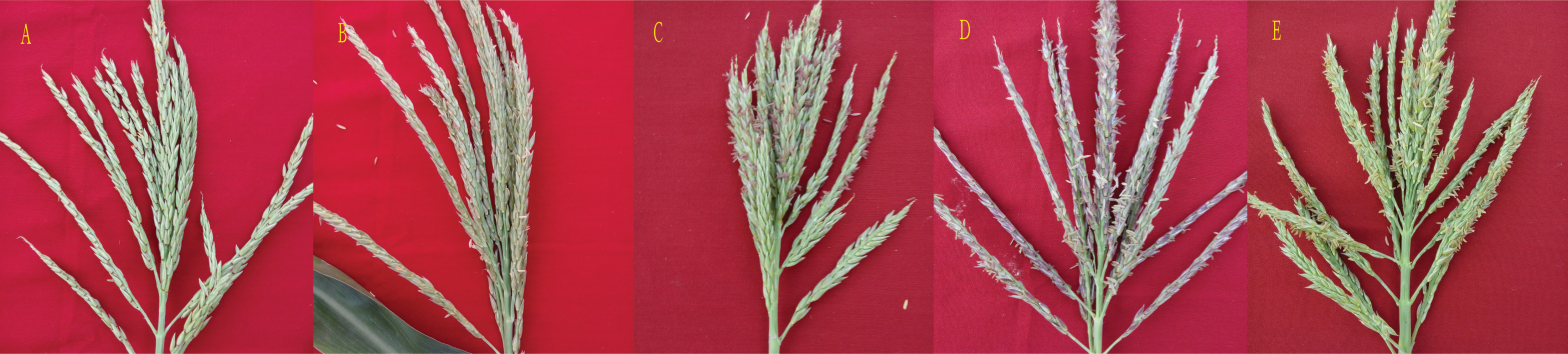
Different male fertility grades of maize anthers. A–E indicated plant fertility grades I–V respectively. I: 0–10% of anthers exerted; II: 11–25% of anthers exerted; III: 26–50% of anthers exerted; IV: 51–75% of anthers exerted; V: over 75% of anthers exerted.

Male fertility of each F_2_ plant during 2013–2015 was graded mainly based on the degree of anther exertion. Anthers fertility were recorded every other day when tiller tassels were starting to branch. Plant male fertility was graded on a scale of I to V as follows (Fig. 1). I: 0–10% of anthers exerted; II: 11–25% of anthers exerted; III: 26–50% of anthers exerted; IV: 51–75% of anthers exerted; V: over 75% of anthers exerted. Plants with scores of I or II were viewed as sterile, while scores of III, IV, and V were recorded as fertile plants (Duvick, 1956; Hu et al., 2006). In addition, we collected each plant pollen from the upper, middle and bottom non-dehiscent anthers on the main stalk to investigate its fertility. Pollen fertility was rated on a scale of 1 to 3 according to the pollen staining ability using 1% (w/v) KI-I_2_: ① <25% stainable pollen; ② 25%–75% pollen stainable; ③ >75% pollen stainable. Any plant with score ① was recorded as sterile and one plant fertility grade was minus 1 if its pollen fertility scored ②. Moreover, during the cultivation of the F_2_ populations at Xishuangbanna in 2014 winter, some normal inbred lines did not shed pollen easily. In view of this, plant fertility belonging to grade II should be assigned to the fertile plants at Xishuangbanna (Chen & Duan, 1986).

### Development and validation of *Rf4* -targeted marker F2/R2

**Figure 2:**
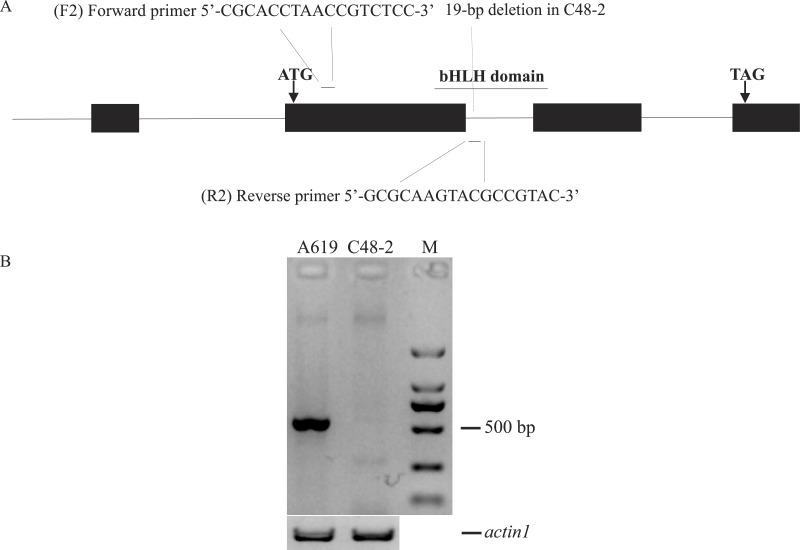
Design and evaluation of the primers F2/R2. (A) Development of F2/R2 primers. There is a 19-bp deletion in male sterile line C48-2 compared with the inbred line A619. (B) Electrophoresis analysis of F2/R2 PCR amplification in A619 and C48-2. “M” was standard molecular weight, “actin1” was taken as positive control.

According to the conservative sequence of GRMZM2G021276_T02, primers F1/R1 (5′-GGAAGGAGGAAACCAAGTCG-3′, 5′-TGTAACGAGCAAGCGGATTTA-3′) were designed to amplify its full length genome sequence. *Rf4* and *rf4* were respectively amplified in A619 and C48-2. As a result, a 19-bp deletion was found in the intron of *rf4* compared to *Rf4* (Fig. S1B). PCR amplification was performed using the following program: initial denaturation at 94 °C for 3 min; 35 cycles of denaturation at 98 °C for 10 s, annealing at 61 °C for 30 s, extension at 68 °C for 2 min 10 s, and a final extension at 68  °C for 5 min. Based on the 19-bp deletion, the primers F2/R2 (5′-CGCACCTAACCGTCTCC-3′, 5′-GCGCAAGTACGCCGTAC-3′) were designed to phenotype *Rf4* and *rf4*. The 5′end of the reverse primer (R2) specifically binds to the region containing the 19bp deletion between *Rf4* and *rf4* (Fig. 2A and Fig. S1B). In order to validate the effectiveness of F2/R2, 30 plants from A619 and C48-2 were used as PCR templates respectively. Genome DNA was extracted from fresh leaves as the modified cetyltrimethylammonium bromide (CTAB) method (Porebski, Bailey & Baum, 1997). PCR amplification was performed using Tsingke Master Mix and the following reaction conditions: initial denaturation at 94 °C for 5 min; 35 cycles of denaturation at 95 °C for 30 s, annealing at 55 °C for 30 s, extension at 72 °C for 45 s, and a final extension at 72 °C for 8 min. The primers F/R (5′-CACCTTCTACAACGAGCTCCG-3′, 5′-TAATCAAGGGCAACGTAGGCA-3′) designed from * actin1* (accession: J01238), which amplified an approximately 500-bp fragment, were used as positive controls (Wang et al., 2008).

Additionally, in order to identify the specificity of primers F2/R2, we got its amplifying band sequence from A619 by direct sequencing and searched it with maizeGDB BLAST program in the maize genome (Andorf et al., 2016).

### Genotypic analysis of (C48-2 × A619) F_2_ by F2/R2

A total of 165 F_2_ plants in 2014 and 150 F_2_ plants in 2015 were used for *Rf4* genotyping. Purified DNA was extracted from fresh leaves following the modified cetyltrimethylammonium bromide (CTAB) method (Porebski, Bailey & Baum, 1997). The presence of an amplification fragment for *Rf4* and no amplification indicated the *rf4* genotype using F2/R2 in this experiment. At the same time, the primers *actin1* F/R (described above) were further examined as positive controls. PCR amplification was performed following the methods described above.

### Allelic analysis of Rf*-A619 and *Rf5*

The plants without *Rf4* in the (C48-2 × A619)F_2_ population were analyzed using the SSR marker bnlg1346 (5′-CATCATGAAGCAATGAAGCC-3′, 5′-CCGCGCCATTATCTAGTTGT-3′), which is tightly linked with the *Rf5* gene (Tang et al., 2001), to identify whether those plants contain *Rf5*. PCR amplification was performed using Tsingke Master Mix and the following reaction conditions: initial denaturation at 94 °C for 5 min; 35 cycles of denaturation at 95 °C for 30 s, annealing at 52 °C for 30 s, extension at 72 °C for 30 s, and a final extension at 72 °C for 8 min.

## Results

### Genetic analysis of fertility restorer gene in A619

In this study, the fertility segregation of (C48-2 × A619) F_2_ have been investigated during 2013–2015 respectively (Table 1 and Table S1). A chi-square test showed that the segregation ratio of male fertile plants to male sterile plants fit 15:1. The statistic analysis results showed that A619 might have two restore genes for C48-2 (Table 1). It is well known that A619 has the restore gene *Rf4*, so we named the other restore gene as Rf*-A619 following the nomenclatural rules of maize genetics (http://www.maizegdb.org/nomenclature).

**Table 1:** Fertility segregations of (C48-2 × A619) F_2_ populations.

Population	Environment	Total plants	Fertile	Sterile	Ratio tested	*χ*^2^	*p*-value
(C48-2 × A619) F_2_	2013, Winter, Xishuangbanna	290	273	17	15:1	0.02	0.88
	2014, Winter, Xishuangbanna	165	151	14	15:1	1.05	0.30
	2015, Spring, Chengdu	150	136	14	15:1	1.94	0.16

### The marker F2/R2 could distinguish the *Rf4* genotype

We developed a marker F2/R2 based on a 19-bp deletion in C48-2 compared with A619 to distinguish the *Rf4* locus genotype (Fig. 2A). As expected, the primers F2/R2 could amplify a fragment of approximately 550-bp in A619, but did not amplify the fragment in the male sterile C48-2 (Fig. 2B). Direct PCR product sequencing results showed the sequence amplified by F2/R2 belonged to a part of *Rf4* (Fig. S2A). Moreover, we found the F2/R2 amplifying sequence could be matched to one unique position in maize genome which is just in gene GRMZM2G021276 (*Rf4*) (Fig. S2B). These results totally confirmed the specificity of the F2/R2 primers. Thus, the amplification fragment by F2/R2 represented for *Rf4_* genotype and the lack of amplification indicated the genotype of *rf4rf4* in this experiment.

### Rf*-A619 exhibited a dominant restorer gene for CMS-C fertility restoration

**Figure 3:**
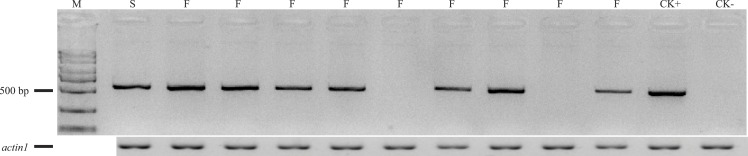
The amplifying products using F2/R2 primers from some (C48-2 × A619) F_2_ individuals. M, standard molecular weight; F, fertile individuals; S, sterile individuals; CK+, DNA from A619 as PCR positive control; CK-, DNA from C48-2 as PCR negative control. Additionally, we took “*actin1*” as positive PCR results to distinguish between no amplification and mistakes of PCR.

The primers F2/R2 were used to genotype *Rf4* in the (C48-2 × A619) F_2_ population, including 165 plants from Xishuangbanna in 2014 and 150 plants from Chengdu in 2015 (Fig. 3). As for the F_2_ population planted at Xishuangbanna in 2014, 126 (*Rf4Rf4* and *Rf4rf4*) out of 165 plants can amplify the fragment, 39 (*rf4rf4*) out of 165 plants cannot amplify the fragments with F2/R2 primers. For the F_2_ population planted at Chengdu in 2015, 116 (*Rf4Rf4* and *Rf4rf4*) out of 150 plants with the PCR products, 34 (*rf4rf4*) out of 150 plants without PCR products by the F2/R2 primers. And furthermore, chi-square test showed with PCR products and without PCR products fitted 3:1 (3/4*Rf4_* and 1/4 *rf4rf4*) ratio that consistent with the heredity pattern of one dominant gene (2014: *χ*^2^ = 0.10, *p* = 0.75; 2015: *χ*^2^ = 0.32, *p* = 0.57) (Table 2). The results gave the answer that the PCR amplification results were reliable and we could conjecture the genotype on the *Rf4* locus through the PCR amplifying results. When combining the fertility investigation of each individual with their F2/R2 amplify results, we found 36 individuals (2014, Xishaungbanna) and 27 individuals (2015, Chengdu) exhibited male fertile but without PCR products by F2/R2 among the F_2_ populations respectively. Moreover, these fertile plants (rf4rf4Rf*-A619_) without F2/R2 PCR fragments accounted for 3/16 of the total F_2_ plants (2014: *χ*^2^ = 0.83, *p* = 0.36; 2015: *χ*^2^ = 0.02, *p* = 0.90). As is known to all, this 3/16 ratio just matched the amount of plants with genotype rf4rf4Rf*-A619Rf*-A619 and rf4rf4Rf*-A619rf*-A619 in the F_2_ population. These above results provided another item of proof in sustaining that the inbred line A619 contains one additional restorer gene for CMS-C fertility restoration besides *Rf4*. Additionally, we also observed plants that had *Rf4*, but were sterile (Table 2), including 11 sterile plants out of 165 plants at Xishuangbanna in 2014 and 7 infertile plants among 150 total plants at Chengdu in 2015.

**Table 2:** Primers F2/R2 amplification results in the (C48-2 × A619) F_2_ population.

Population	Total plants	With PCR product	Fertility	Without PCR product	Fertility
(C48-2 × A619) F_2_	165	126	115 Fertile	39	36 Fertile
			11 Sterile		3 Sterile
	150	116	109 Fertile	34	27 Fertile
			7 Sterile		7 Sterile

### Rf*-A619 is not allelic to *Rf5*

**Figure 4:**
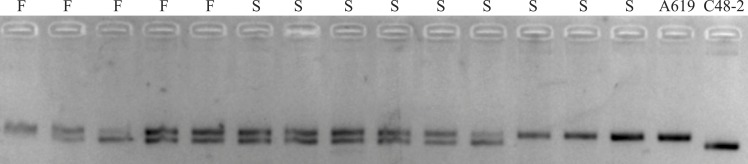
SSR products amplified using bnlg1346 from parents and F_2_ individuals without *Rf4*. S, a subset of F_2_ sterile individuals; F, a subset of F_2_ fertile individuals.

In order to verify whether the additional restore gene in A619 is allelic to *Rf5* for CMS-C, the SSR marker bnlg1346 tightly linked to *Rf5* was used for the individuals without F2/R2 products. Polymorphism amplifying bands were detected between C48-2 and A619 (Fig. 4). However, no evidence of plants fertility co-segregation to the SSR amplifying fragments was found. A fragment amplified in A619, can also be amplified in the male sterile individuals. The bnlg1346 marker amplification results indicated that the male fertile individuals without F2/R2 amplification in F_2_ populations could not be restored by *Rf5*, so it is concluded that the additional restore gene Rf*-A619 is not allelic to *Rf5*.

## Discussion

Up to now, only two dominant restorer genes (*Rf4* and *Rf5*) and some partial fertility restorer genes for CMS-C in maize have been discovered. Insufficient main restorer genes and restorer lines limit the use of CMS-C in hybrid seed production (Stevanovic et al., 2016). Thus, discovering and mapping new restorer genes are of great importance. In the present study, we identified a novel dominant restorer gene Rf*-A619 in the inbred line A619 for CMS-C through genetic analyses and molecular markers. In the future, it is imperative to identify and clone Rf*-A619.

In contrast to our results, previous findings indicate that A619 only contains one single restorer gene (Chen, Luo & Ji, 1979; Tang et al., 2001). Therefore, it is of great interest to explain why Rf*-A619 does not rescue some c-type sterile lines in maize. Similar events have also occurred in other inbred lines. According to various studies of CMS-C fertility restoration (Chen & Chen, 1989; Chen & Duan, 1986; Chen, Luo & Ji, 1979; Huang et al., 1997; Tang et al., 2001), when crossed with CMS-Chuangzaosi and CMS-Cernan24, Fengke1 (*Rf4Rf4Rf5Rf5*) seems to have only one restorer gene, but it appears to have two loci for CMS-CMO17 and CMS-C237. Similarly, Guang10-2 (*Rf4Rf4rf5rf5*) exhibited one restorer gene in CMS-Chuangzaosi and CMS-Cernan24, but two restorer genes were observed in progeny from crosses with CMO17. Most interestingly, parallel phenomena were reported in rice. The same restorer line rescued CMS via different numbers of restorer genes when facing different male sterile lines with the same cytoplasm (Zhou et al., 1986; Zhu, Duan & Deng, 2001). The results of this study, together with those of previous related reports indicated that the functions of restorer genes are affected by the genetic backgrounds of sterile lines. Moreover, in the present experiment, it was surprised that there were some sterile plants with *Rf4* genotype. Some studies (Tang et al., 2001; Ren et al., 2012) have indicated that an inbred line containing *Rf4* is capable of completely rescuing CMS-C fertility. However, Kohls et al. (2011) found that most fertile plants exhibit partial restoration in the (B37C × K55) F_2_ population, though the inbred line K55 contains *Rf4*. Some studies suggested that the interactions of some factors between male parent and female parent might also contribute to plant cytoplasmic male fertility restoration (Sharma, Singh & Singh, 2005; Sotchenko, Gorbacheva & Kosogorova, 2007). The above studies combining with our results indicated that different parents might have effects on the *Rf4* function. In the future, it will be necessary to determine the fertility restoration mechanism of *Rf4* to ensure its normal function and to increase its use in hybrid seed production.

##  Supplemental Information

10.7717/peerj.2719/supp-1 Table S1 The raw data of genotype and phenotype of the (C48-2 ttimes A619) F_2_ populaion at Xishuangbanna in 2014 and Chengdu in 2015 For genotype, 1 indicated having PCR product, 0 represented for no PCR product by primers F2/R2. For phenotype, F: fertile individual, S: sterile individual. The number indicated the serial number of plants.

10.7717/peerj.2719/supp-2 Figure S1 PCR amplification and sequencing of *Rf4* with F1/R1 in C48-2 and A619 (A) Electrophoresis analysis of F1/R1 PCR amplification. (B) Comparison of the genome sequence of *Rf4* between A619 and C48-2. The red letters indicated the sequence of primers F2/R2. The underlined regions are the amplifying fragment by primers F2/R2.

10.7717/peerj.2719/supp-3 Figure S2 Confirmation of the specificity of primers F2/R2 (A) Comparison of the *Rf4* sequence in A619 and F2/R2 amplifying sequence. A619-*Rf4* indicated the genome sequence of *Rf4* in the inbred line A619 and the number in it represent for bases position. (B) The search result of F2/R2 amplifying sequence in maize genome. The red bar reprsented for the position of F2/R2 amplifying sequence in maize B73 genome.

## Additional Information and Declarations

### Data Availability

The following information was supplied regarding data availability: The raw data has been supplied as a Supplementary File.
