# Supplementary material for: A preliminary identification of Rf*-A619, a novel restorer gene for CMS-C in maize (Zea mays L.)
Source: PeerJ. 2016 Nov 22;4:e2719. doi: 10.7717/peerj.2719 (PMC5126625; doi:10.7717/peerj.2719)
Supplement: Table S1 — For genotype, 1 indicated having PCR product, 0 represented for no PCR product by primers F2/R2. For phenotype, F: fertile individual, S: sterile individual. The number indicated the serial number of plants. [file peerj-04-2719-s001.docx]

**Table S1 The raw data of genotype and phenotype of the (C48-2×A619) F_2_ populaion at Xishuangbanna in 2014 and Chengdu in 2015.**

| NO. | Genotype | Fertility grade | Phenotype | No. | Genotype | Fertility grade | Phenotype | No. | Genotype | Fertility grade | Phenotype |
| --- | --- | --- | --- | --- | --- | --- | --- | --- | --- | --- | --- |
| 14-1 | 0 | Ⅲ | F | 14-150 | 1 | Ⅳ | F | 15-46 | 1 | Ⅴ | F |
| 14-2 | 1 | Ⅳ | F | 14-151 | 1 | Ⅰ | S | 15-47 | 1 | Ⅴ | F |
| 14-3 | 1 | Ⅲ | F | 14-152 | 0 | Ⅳ | F | 15-48 | 1 | Ⅲ | F |
| 14-6 | 1 | Ⅳ | F | 14-153 | 1 | Ⅲ | F | 15-49 | 0 | Ⅴ | F |
| 14-9 | 1 | Ⅱ | F | 14-154 | 0 | Ⅲ | F | 15-50 | 1 | Ⅴ | F |
| 14-10 | 1 | Ⅲ | F | 14-155 | 1 | Ⅱ | F | 15-51 | 1 | Ⅴ | F |
| 14-13 | 1 | Ⅳ | F | 14-156 | 1 | Ⅱ | F | 15-52 | 1 | Ⅴ | F |
| 14-15 | 0 | Ⅳ | F | 14-157 | 1 | Ⅳ | F | 15-53 | 1 | Ⅴ | F |
| 14-16 | 1 | Ⅲ | F | 14-158 | 0 | Ⅳ | F | 15-54 | 0 | Ⅴ | F |
| 14-17 | 1 | Ⅳ | F | 14-162 | 1 | Ⅳ | F | 15-55 | 1 | Ⅴ | F |
| 14-18 | 1 | Ⅲ | F | 14-163 | 1 | Ⅲ | F | 15-56 | 0 | Ⅴ | F |
| 14-19 | 0 | Ⅳ | F | 14-164 | 0 | Ⅱ | F | 15-57 | 1 | Ⅴ | F |
| 14-20 | 0 | Ⅰ | S | 14-165 | 1 | Ⅲ | F | 15-58 | 1 | Ⅲ | F |
| 14-21 | 1 | Ⅰ | S | 14-166 | 1 | Ⅲ | F | 15-59 | 0 | Ⅴ | F |
| 14-23 | 1 | Ⅲ | F | 14-169 | 0 | Ⅳ | F | 15-60 | 1 | Ⅴ | F |
| 14-24 | 1 | Ⅳ | F | 14-170 | 1 | Ⅲ | F | 15-61 | 1 | Ⅴ | F |
| 14-25 | 0 | Ⅲ | F | 14-173 | 0 | Ⅳ | F | 15-62 | 1 | Ⅴ | F |
| 14-27 | 1 | Ⅰ | S | 14-177 | 1 | Ⅲ | F | 15-63 | 1 | Ⅱ | S |
| 14-30 | 1 | Ⅰ | S | 14-182 | 1 | Ⅳ | F | 15-64 | 1 | Ⅴ | F |
| 14-31 | 1 | Ⅳ | F | 14-183 | 1 | Ⅳ | F | 15-65 | 1 | Ⅳ | F |
| 14-32 | 1 | Ⅳ | F | 14-185 | 1 | Ⅲ | F | 15-66 | 1 | Ⅴ | F |
| 14-34 | 1 | Ⅳ | F | 14-186 | 1 | Ⅳ | F | 15-67 | 0 | Ⅳ | F |
| 14-35 | 0 | Ⅳ | F | 14-187 | 1 | Ⅳ | F | 15-68 | 1 | Ⅴ | F |
| 14-36 | 1 | Ⅲ | F | 14-189 | 1 | Ⅰ | S | 15-69 | 1 | Ⅴ | F |
| 14-39 | 1 | Ⅲ | F | 14-190 | 1 | Ⅳ | F | 15-70 | 1 | Ⅴ | F |
| 14-41 | 1 | Ⅰ | S | 14-191 | 0 | Ⅳ | F | 15-71 | 0 | Ⅴ | F |
| 14-42 | 1 | Ⅲ | F | 14-194 | 0 | Ⅲ | F | 15-72 | 0 | Ⅴ | F |
| 14-44 | 0 | Ⅲ | F | 14-196 | 1 | Ⅳ | F | 15-73 | 0 | Ⅴ | F |
| 14-45 | 1 | Ⅰ | S | 14-197 | 1 | Ⅳ | F | 15-74 | 0 | Ⅰ | S |
| 14-46 | 1 | Ⅲ | F | 14-198 | 1 | Ⅳ | F | 15-75 | 1 | Ⅲ | F |
| 14-47 | 1 | Ⅳ | F | 14-199 | 1 | Ⅲ | F | 15-76 | 1 | Ⅴ | F |
| 14-48 | 1 | Ⅴ | F | 14-200 | 1 | Ⅴ | F | 15-77 | 1 | Ⅴ | F |
| 14-49 | 1 | Ⅲ | F | 14-201 | 1 | Ⅲ | F | 15-78 | 1 | Ⅴ | F |
| 14-50 | 1 | Ⅳ | F | 14-202 | 1 | Ⅲ | F | 15-79 | 1 | Ⅴ | F |
| 14-51 | 1 | Ⅳ | F | 14-205 | 1 | Ⅰ | S | 15-80 | 0 | Ⅴ | F |
| 14-52 | 1 | Ⅲ | F | 14-206 | 1 | Ⅳ | F | 15-81 | 1 | Ⅴ | F |
| 14-54 | 0 | Ⅳ | F | 14-207 | 1 | Ⅲ | F | 15-82 | 1 | Ⅴ | F |
| 14-55 | 1 | Ⅳ | F | 14-208 | 0 | Ⅳ | F | 15-83 | 0 | Ⅴ | F |
| 14-56 | 1 | Ⅳ | F | 14-212 | 0 | Ⅳ | F | 15-84 | 1 | Ⅴ | F |
| 14-57 | 1 | Ⅳ | F | 14-214 | 1 | Ⅳ | F | 15-85 | 1 | Ⅴ | F |
| 14-58 | 1 | Ⅲ | F | 14-215 | 1 | Ⅳ | F | 15-86 | 1 | Ⅴ | F |
| 14-59 | 1 | Ⅱ | F | 14-216 | 1 | Ⅳ | F | 15-87 | 1 | Ⅴ | F |
| 14-60 | 1 | Ⅲ | F | 14-217 | 1 | Ⅳ | F | 15-88 | 1 | Ⅴ | F |
| 14-61 | 1 | Ⅴ | F | 14-220 | 1 | Ⅴ | F | 15-89 | 1 | Ⅲ | F |
| 14-62 | 1 | Ⅳ | F | 14-221 | 1 | Ⅳ | F | 15-90 | 1 | Ⅴ | F |
| 14-63 | 0 | Ⅴ | F | 14-222 | 1 | Ⅲ | F | 15-91 | 1 | Ⅳ | F |
| 14-64 | 0 | Ⅲ | F | 14-223 | 1 | Ⅳ | F | 15-92 | 1 | Ⅴ | F |
| 14-67 | 1 | Ⅲ | F | 14-224 | 1 | Ⅳ | F | 15-93 | 1 | Ⅴ | F |
| 14-70 | 0 | Ⅰ | S | 14-225 | 1 | Ⅳ | F | 15-94 | 1 | Ⅳ | F |
| 14-71 | 1 | Ⅳ | F | 14-226 | 1 | Ⅴ | F | 15-95 | 1 | Ⅴ | F |
| 14-73 | 1 | Ⅴ | F | 14-227 | 1 | Ⅳ | F | 15-96 | 1 | Ⅲ | F |
| 14-74 | 1 | Ⅳ | F | 14-228 | 0 | Ⅳ | F | 15-97 | 1 | Ⅳ | F |
| 14-75 | 1 | Ⅳ | F | 14-229 | 1 | Ⅲ | F | 15-98 | 1 | Ⅱ | S |
| 14-76 | 1 | Ⅰ | S | 14-230 | 1 | Ⅲ | F | 15-99 | 0 | Ⅰ | S |
| 14-78 | 1 | Ⅳ | F | 14-235 | 1 | Ⅳ | F | 15-100 | 1 | Ⅴ | F |
| 14-79 | 1 | Ⅳ | F | 14-236 | 1 | Ⅲ | F | 15-101 | 1 | Ⅴ | F |
| 14-80 | 1 | Ⅳ | F | 14-237 | 1 | Ⅳ | F | 15-102 | 1 | Ⅴ | F |
| 14-81 | 1 | Ⅰ | S | 14-238 | 0 | Ⅳ | F | 15-103 | 1 | Ⅴ | F |
| 14-83 | 1 | Ⅳ | F | 14-239 | 1 | Ⅳ | F | 15-104 | 0 | Ⅴ | F |
| 14-86 | 1 | Ⅲ | F | 14-240 | 1 | Ⅴ | F | 15-105 | 1 | Ⅴ | F |
| 14-87 | 1 | Ⅲ | F | 15-1 | 1 | Ⅴ | F | 15-106 | 1 | Ⅴ | F |
| 14-88 | 0 | Ⅳ | F | 15-2 | 1 | Ⅴ | F | 15-107 | 0 | Ⅴ | F |
| 14-89 | 1 | Ⅳ | F | 15-3 | 1 | Ⅳ | F | 15-108 | 1 | Ⅴ | F |
| 14-91 | 0 | Ⅲ | F | 15-4 | 1 | Ⅴ | F | 15-109 | 1 | Ⅴ | F |
| 14-93 | 1 | Ⅳ | F | 15-5 | 1 | Ⅴ | F | 15-110 | 0 | Ⅰ | S |
| 14-94 | 1 | Ⅳ | F | 15-6 | 1 | Ⅴ | F | 15-111 | 0 | Ⅴ | F |
| 14-95 | 0 | Ⅳ | F | 15-7 | 1 | Ⅴ | F | 15-112 | 1 | Ⅴ | F |
| 14-97 | 1 | Ⅳ | F | 15-8 | 1 | Ⅴ | F | 15-113 | 1 | Ⅴ | F |
| 14-98 | 1 | Ⅳ | F | 15-9 | 1 | Ⅴ | F | 15-114 | 1 | Ⅰ | S |
| 14-99 | 1 | Ⅲ | F | 15-10 | 1 | Ⅲ | F | 15-115 | 1 | Ⅳ | F |
| 14-100 | 1 | Ⅲ | F | 15-11 | 1 | Ⅴ | F | 15-116 | 1 | Ⅴ | F |
| 14-104 | 1 | Ⅳ | F | 15-12 | 1 | Ⅴ | F | 15-117 | 0 | Ⅲ | F |
| 14-105 | 1 | Ⅳ | F | 15-13 | 1 | Ⅴ | F | 15-118 | 0 | Ⅴ | F |
| 14-106 | 1 | Ⅱ | F | 15-14 | 1 | Ⅱ | S | 15-119 | 0 | Ⅴ | F |
| 14-108 | 1 | Ⅳ | F | 15-15 | 0 | Ⅴ | F | 15-120 | 0 | Ⅴ | F |
| 14-109 | 0 | Ⅳ | F | 15-16 | 1 | Ⅴ | F | 15-121 | 1 | Ⅳ | F |
| 14-110 | 1 | Ⅱ | F | 15-17 | 1 | Ⅲ | F | 15-122 | 1 | Ⅳ | F |
| 14-111 | 0 | Ⅳ | F | 15-18 | 0 | Ⅰ | S | 15-123 | 1 | Ⅴ | F |
| 14-112 | 0 | Ⅲ | F | 15-19 | 1 | Ⅴ | F | 15-124 | 1 | Ⅴ | F |
| 14-114 | 1 | Ⅲ | F | 15-20 | 1 | Ⅴ | F | 15-125 | 1 | Ⅴ | F |
| 14-115 | 0 | Ⅳ | F | 15-21 | 1 | Ⅴ | F | 15-126 | 1 | Ⅰ | S |
| 14-116 | 0 | Ⅳ | F | 15-22 | 1 | Ⅴ | F | 15-127 | 1 | Ⅲ | F |
| 14-117 | 1 | Ⅳ | F | 15-23 | 1 | Ⅴ | F | 15-128 | 1 | Ⅲ | F |
| 14-120 | 1 | Ⅲ | F | 15-24 | 1 | Ⅴ | F | 15-129 | 1 | Ⅳ | F |
| 14-121 | 0 | Ⅳ | F | 15-25 | 1 | Ⅴ | F | 15-130 | 1 | Ⅴ | F |
| 14-122 | 1 | Ⅲ | F | 15-26 | 1 | Ⅴ | F | 15-131 | 1 | Ⅲ | F |
| 14-124 | 1 | Ⅰ | S | 15-27 | 1 | Ⅴ | F | 15-132 | 1 | Ⅳ | F |
| 14-126 | 1 | Ⅲ | F | 15-28 | 0 | Ⅴ | F | 15-133 | 1 | Ⅳ | F |
| 14-128 | 1 | Ⅳ | F | 15-29 | 0 | Ⅴ | F | 15-134 | 1 | Ⅲ | F |
| 14-130 | 0 | Ⅲ | F | 15-30 | 0 | Ⅴ | F | 15-135 | 0 | Ⅰ | S |
| 14-131 | 0 | Ⅳ | F | 15-31 | 1 | Ⅴ | F | 15-136 | 1 | Ⅴ | F |
| 14-133 | 0 | Ⅲ | F | 15-32 | 1 | Ⅴ | F | 15-137 | 1 | Ⅴ | F |
| 14-135 | 1 | Ⅲ | F | 15-33 | 1 | Ⅴ | F | 15-138 | 0 | Ⅴ | F |
| 14-136 | 1 | Ⅳ | F | 15-34 | 1 | Ⅳ | F | 15-139 | 1 | Ⅳ | F |
| 14-137 | 1 | Ⅳ | F | 15-35 | 1 | Ⅴ | F | 15-140 | 1 | Ⅳ | F |
| 14-138 | 0 | Ⅲ | F | 15-36 | 1 | Ⅴ | F | 15-141 | 1 | Ⅴ | F |
| 14-139 | 1 | Ⅲ | F | 15-37 | 0 | Ⅴ | F | 15-142 | 1 | Ⅰ | S |
| 14-141 | 1 | Ⅲ | F | 15-38 | 1 | Ⅴ | F | 15-143 | 0 | Ⅴ | F |
| 14-142 | 1 | Ⅲ | F | 15-39 | 1 | Ⅴ | F | 15-144 | 1 | Ⅴ | F |
| 14-143 | 1 | Ⅳ | F | 15-40 | 1 | Ⅴ | F | 15-145 | 0 | Ⅰ | S |
| 14-144 | 1 | Ⅲ | F | 15-41 | 1 | Ⅴ | F | 15-146 | 0 | Ⅴ | F |
| 14-145 | 0 | Ⅳ | F | 15-42 | 0 | Ⅴ | F | 15-147 | 0 | Ⅴ | F |
| 14-146 | 0 | Ⅰ | S | 15-43 | 1 | Ⅴ | F | 15-148 | 1 | Ⅴ | F |
| 14-147 | 1 | Ⅱ | F | 15-44 | 1 | Ⅴ | F | 15-149 | 1 | Ⅰ | S |
| 14-149 | 0 | Ⅱ | F | 15-45 | 0 | Ⅰ | S | 15-150 | 1 | Ⅲ | F |

For genotype, 1 indicated having PCR product, 0 represented for no PCR product by primers F2/R2. For phenotype, F: fertile individual, S: sterile individual. The number indicated the serial number of plants.
