# Supplementary material for: A preliminary identification of Rf*-A619, a novel restorer gene for CMS-C in maize (Zea mays L.)
Source: PeerJ. 2016 Nov 22;4:e2719. doi: 10.7717/peerj.2719 (PMC5126625; doi:10.7717/peerj.2719)
Supplement: Figure S1 — (A) Electrophoresis analysis of F1/R1 PCR amplification. (B) Comparison of the genome sequence of Rf4 between A619 and C48-2. The red letters indicated the sequence of primers F2/R2. The underlined regions are the amplifying fragment by primers F2/R2. [file peerj-04-2719-s002.pdf]

Figure S1 PCR amplification and sequencing of *Rf4* with F1/R1 in C48-2 and A619. (A) Electrophoresis analysis of F1/R1 PCR amplification. (B) Comparison of the genome sequence of *Rf4* between A619 and C48-2. The red letters indicated the sequence of primers F2/R2. The underlined regions are the amplifying fragment by primers F2/R2.

S1A

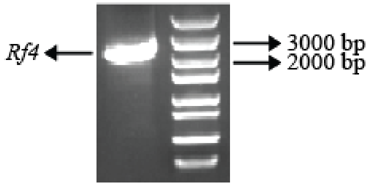

S1B

```
C48-2    CTACACTACACACACATATACATGGGCAACGCAAGGCCACCTTTCTGAATCCTGCATGAGCGTGTACCACTAGAATTGTCAGTGTGTGCGGTGTATGGCA   100
A619     CTACACTACACACACATATACATGGGCAACGCAAGGCCACCTTTCTGAATCCTGCATGAGCGTGTACCACTAGAATTGTCAGTGTGTGCGGTGTATGGCA   100
Consensuctacactacacacacatatacatgggcaacgcaaggccacctttctgaatcctgcatgagcgtgtaccactagaattgtcagtgtgtgcggtgtatggca

C48-2    GGTTTTTGGTTCGGCAAGTGGGGCCCTCCGGGGAGGAATCTCAGTAACAAACCGCTCTTCTGAAAAGGTCAGCCATCCCCGGTCCGGTCCGGTGATGTCG   200
A619     GGTTTTTGGTTCGGCAAGTGGGGCCCTCCGGGGAGGAATCTCAGTAACAAACCGCTCTTCTGAAAAGGTCAGCCATCCCCGGTCCGGTCCGGTGATGTCG   200
Consensuggtttttggttcggcaagtggggccctccggggaggaatctcagtaacaaaccgctcttctgaaaaggtcagccatccccggtccggtccggtgatgtcg

C48-2    TCGCTGTCGCTCTGCTAGCTTGCTGCCGATCCCCCCCCCCCCCCCTTCTTCTCTCTACCCCTCCCTCCACCTCATAAATACTTAGTTTAATAACCTTGC   300
A619     TCGCTGTCGCTCTGCTAGCTTGCTGCCGATCCACACCCCCTCTCCCTTCTTCTCTCTACCCCTCCCTCCACCTCATAAATACTTAGTTTAATAACCTTGC   300
Consensutcgctgtcgctctgctagcttgctgccgatcc c ccccc c cccttcttctctctacccctccctccacctcataaatacttagtttaataaccttgc

C48-2    ACTGCCGCAGTAGCCCTTAACTGCTGCT....ATCTATCTCTTTTCTGAAGGAAAAAAAAGGTTTGATACTCCTCTACCTAGCTAGTCCTGCATGCCGCT   396
A619     ACTGCCGCAGTAGCCCTTAACTGCTGCTATATATCTATCTCTTTTCTGAAGGAAAAAAAAGGTTTGATACTCCTCTAGCTAGCTAGTCCTGCATGCCGCT   400
Consensuactgccgcagtagcccttaactgctgct    atctatctcttttctgaaggaaaaaaaaggtttgatactcctcta ctagctagtcctgcatgccgct

C48-2    AATGTGCGTCTTGCCTGTTTATTTGTTCTTAATAAGGGCTGCCTATCTATTATATTTTGCACCTGTTTTGCTGTGTTCTTGGTAACTAGCTTAATTCCTT   496
A619     AATGTTCGTCTTGCCTGTTTATTTGTTCTTAATAAGGGCTGCCTATCTATTATATTTTGCACCTGTTTTGCTGTGTTCTTGGTAACTAGCTTAATTCCTT   500
Consensuaatgt cgtcttgcctgtttatttgttcttaataagggctgcctatctattatattttgcacctgttttgctgtgttcttggtaactagcttaattcctt

C48-2    TGCCTACAATCGTCAAATCCCCCCCATCATCAGTCAGATGAACTTTTGATCGAATTGAAGTTGTTCTTCTAATTCGGCCCCAGCAGCGCCCATGCATCTG   596
A619     CGCCTACAATCGTCAAATCCCCCCCATCATCAGTCAGATGAACTTTTGATCGAATTGAAGTTGTTCTTCTAATTCGGCCCCAGCAGCGCCCATGCATCTG   600
Consensusgcctacaatcgtcaaatcccccccatcatcagtcagatgaacttttgatcgaattgaagttgttcttctaattcggccccagcagcgcccatgcatctg

C48-2    GTTTTATTTGCTTTCTGTTGGGTATAATATGCAAGACCTTTTGTTGCTAGGGCAAGGCTGCAACCACATGCGTGTACTGAACTCATGATGTAACTCATCC   696
A619     GTTTTATTTGCTTTCTGTTGGGTATAATATGCAAGACCTTTTGTTGCTAGGGCAAGGCTGCAACCACATGCGTGTACTGAACTCATGATGTAACTCATCC   700
Consensugttttatttgctttctgttgggtataatatgcaagaccttttgttgctagggcaaggctgcaaccacatgcgtgtactgaactcatgatgtaactcatcc

C48-2    TTTTTGTTTGCTCACAGAATCACTACTCTACTGCACTTCCTTTTCATCCGATCCGCAATCTTTTTTTTCTTTTACATGCTTTAGTTTTCTCTCTTTCTTG   796
A619     TTTTTGTTTGCTCACAGAATCACTACTCTACTGCACTTCCTTTTCATCCGATCCGCAATCTTTTTTTTCTTTTACATGCTTTAGTTTTCTCTCTTTCTTG   800
Consensutttttgtttgctcacagaatcactactctactgcacttccttttcatccgatccgcaatcttttttttcttttacatgctttagttttctctctttcttg

C48-2    ATTACAAACATGATTACTGGAACTTTCTTAGGCTGCCTTCCCCTTCCTTGGATCTGCTTTAGTTTTCTTTTTTGGGCTACCGCGCGCGGCTTATTTGAGT   896
A619     ATTACAAACATGATTACTGGAACTTTCTTAGGCTGCCTTCCCCTTCCTTGGATCTGCTTTAGTTTTCTTTTTGGGGCTACCGCGCGCGGCTTATTTGAGT   900
Consensuattacaaacatgattactggaactttcttaggctgccttccccttccttggatctgctttagttttcttttt gggctaccgcgcgcggcttatttgagt

C48-2    TTATCACTTGCTGCATATACATAATATATATATACATGCATGCGATGGCGTTCATGTTACTCAACTACAGATCTGTTTCTGTTCGTGTGTTTCAGTTCAG   996
A619     TTATCACTTGCTGCATATACATAATATATATATACATGCATGCGATGGCGTTCATGTTACTCAACTACAGATCTGTTTCTGTTCGTGTGTTTCAGTTCAG  1000
Consensuttatcacttgctgcatatacataatatatatatacatgcatgcgatggcgttcatgttactcaactacagatctgtttctgttcgtgtgtttcagttcag

C48-2    CGCGCAGTTAAGCATAGCAGGACGACCACGACGATGTATCACCCGCAGTGCGAGCTCCTGACGATGGCGCACGAAACGCCGGACCTGGACGCCGGCCAGC  1096
A619     CGCGCAGTTAAGCATAGCAGGACGACCACGACGATGTATCACCCGCAGTGCGAGCTCCTGACGATGGCGCACGAAACGCCGGACCTGGACGCCGGCCAGC  1100
Consensucgcgcagttaagcatagcaggacgaccacgacgatgtatcacccgcagtgcgagctcctgacgatggcgcacgaaacgccggacctggacgccggccagc
  F2 5' -CGCACCTAACCGTCTCC-3'
C48-2    CGCACCTAACCGTCTCCGGCGTCGCCAGCATCCCGGCAGAGCTGAGCTTCCACCTGCTGCACTCGCTCGACGCCGCGGCGGCGGTCAATCCCGTCACGGC  1196
A619     CGCACCTAACCGTCTCCGGCGTCGCCAGCATCCCGGCAGAGCTGAGCTTCCACCTGCTGCACTCGCTCGACGCCGCGGCGGCGGTCAATCCCGTCACGGC  1200
Consensucgcacctaaccgtctccggcgtcgccagcatcccggcagagctgagcttccacctgctgcactcgctcgacgccgcggcggcggtcaatcccgtcacggc

C48-2    GCCGCCGCAGTCCACCATCGACTACTTCCTCGGCGGCGCCGATCCCCACCAGCAGGCCATGCAGTACGAGCCGCTGCCGCCCGCCGCGGGCGGCCACCAC  1296
A619     GCCGCCGCAGTCCACCATCGACTACTTCCTCGGCGGCGCCGATCCCCACCAGCAGGCCATGCAGTACGAGCCGCTGCCGCCCGCCGCGGGCGGCCACCAC  1300
Consensugccgccgcagtccaccatcgactacttcctcggcggcgccgatccccaccagcaggccatgcagtacgagccgctgccgcccgccgcgggcggccaccac

C48-2    CAGTACACCATGGACATGTTCCGCGACTACTGCGACGGCCACTACCCCACCGCCGAGCCGTACATCCGCGGGACAATGACTGGAGCCCTCGTGTTCGGGG  1396
A619     CAGTACACCATGGACATGTTCCGCGACTACTGCGACGGCAACTACCCCACCGCCGAGCCGTACATCCGCGGGACAATGACTGGAGCCCTCGTGTTCGGGG  1400
Consensucagtacaccatggacatgttccgcgactactgcgacggc actaccccaccgccgagccgtacatccgcgggacaatgactggagccctcgtgttcgggg

C48-2    CCACCGACGACGACGACTCGGCCGCTGCCTACATGCCCGGGGGGCACTTTGAGACCTCCCCGCCGCCGCCACGCGCCACCGGCCGCGGCAGGAAGCGGGG  1496
A619     CCACCGACGACGACGACTCGGCCGCTGCCTACATGCCCGGGGGGCACTTTGAGACCTCCCCGCCGCCGCCACGCGCCACCGGCCGCGGCAGGAAGCGGGG  1500
Consensuccaccgacgacgacgactcggccgctgcctacatgcccggggggcactttgagacctccccgccgccgccacgcgccaccggccgcggcaggaagcgggg

C48-2    CAGGGCGCTGGGCGGCGGCTTCCATGCTGTGCTGGCCAACGGCGTCGAGAAGAAGGAGAAGCAGCGCCGGCTGCGGCTCACCGAGAAGTTTACGGCCCTC  1596
A619     CAGGGCGCTGGGCGGCGGCTTCCATGCTGTGCTGGCCAACGGCGTCGAGAAGAAGGAGAAGCAGCGCCGGCTGCGGCTCACCGAGAAGTTTACGGCCCTC  1600
Consensucagggcgctgggcggcggcttccatgctgtgctggccaacggcgtcgagaagaaggagaagcagcgccggctgcggctcaccgagaagtttacggccctc

C48-2    ATGCACCTCATACCCAACGTTACGAAGGTCGTAC...................CAAATCCTCCTCTTATGTTCG...TCCATCGTCTCAAATTAATTCAC  1674
A619     ATGCACCTCATACCCAACGTTACGAAGGTCGTACGGCGTACTTGCGCGCGGACCAAATCCTCCTCTTATGTTCGTCGTCCATCGTCTCAAATTAATTCAC  1700
Consensuatgcacctcatacccaacgttacgaaggtcgtac                   caaatcctcctcttatgttcg   tccatcgtctcaaattaattcac
                                       R2 3' -CATGCCGCATGAACGCG-5'
C48-2    GGTTCTTGTTGTTGTTTATTTTTTGCGCACTGCAGACTGATAGGGCGACGGTGATCTCGGACGCGATCGAGTACATCCAGGAGCTGGGGAGGACGGTGGA  1774
A619     GGTTCTTGTTGTTGTTTATTTTTTGCGCACTGCAGACTGATAGGGCGACGGTGATCTCGGACGCGATCGAGTACATCCAGGAGCTGGGGAGGACGGTGGA  1800
Consensuggttcttgttgttgtttattttttgcgcactgcagactgatagggcgacggtgatctcggacgcgatcgagtacatccaggagctggggaggacggtgga

C48-2    GGAGCTGACGCTGCTGGTGGAGAAGAAGCGGCGCCGGAGGGAGCTGCAGGGGGACGTCGTGGACGCGGCGCCGGCTGCGGTGGTTGCTGCCGCCGGTGAG  1874
A619     GGAGCTGACGCTGCTGGTGGAGAAGAAGCGGCGCCGGAGGGAGCTGCAGGGGGACGTCGTGGACGCGGCGCCGGCTGCGGTGGTTGCTGCCGCCGGTGAG  1900
Consensuggagctgacgctgctggtggagaagaagcggcgccggagggagctgcagggggacgtcgtggacgcggcgccggctgcggtggttgctgccgccggtgag

C48-2    GCGGAGAGCTCGGAGGGCGAGGTGGCTCCTCCGCCGCCGGCCGTGCCGCGGCAGCCGATCCGGAGCACGTACATCCAGCGGCGGAGCAAGGACACGTCCG  1974
A619     GCGGAGAGCTCGGAGGGCGAGGTGGCTCCTCCGCCGCCGGCCGTGCCGCGGCAGCCGATCCGCAGCACGTACATCCAGCGGCGGAGCAAGGACACGTCCG  2000
Consensugcggagagctcggagggcgaggtggctcctccgccgccggccgtgccgcggcagccgatccg agcacgtacatccagcggcggagcaaggacacgtccg

C48-2    TGGACGTGCGGATCGTGGAGGAGGACGTGAACATCAAGCTCACCAAGCGCCGGCGCGACGGGTGCCTCGCAGCCGCGTCGCGCGCGCTGGATGACCTCCG  2074
A619     TGGACGTGCGGATTGTGGAGGAGGACGTGAACATCAAGCTCACCAAGCGCCGGCGCGACGGGTGCCTCGCAGCCGCGTCGCGCGCGCTGGACGACCTCCG  2100
Consensutggacgtgcggat gtggaggaggacgtgaacatcaagctcaccaagcgccggcgcgacgggtgcctcgcagccgcgtcgcgcgcgctgga gacctccg

C48-2    CCTTGACCTCGTCCACCTCTCCGGCGGCAAGATCGGTGACTGTCAAATCTACATGTTCAACACCAAGGTACATACGAATACGATACGTAGCCATTGATCG  2174
A619     CCTTGACCTCGTCCACCTCTCCGGCGGCAAGATCGGTGACTGTCAAATCTACATGTTCAACACCAAGGTACATACGAATACGATAGGTAACCATTGATCG  2200
Consensuccttgacctcgtccacctctccggcggcaagatcggtgactgtcaaatctacatgttcaacaccaaggtacatacgaatacgata gta ccattgatcg

C48-2    ATCTGTAATTCTGTAGCCTGACGATT...............CCGAGGTTTCTG....................GTGCTAAAAAATGCATCTTTTTTTC.T  2238
A619     ATCTGTAATTCTGTAGCCTGACGATTTCATGCATTACTTTTCCGAGGTTTCTGTGCTATACTACCTAATCTAGGTGCTAAAAAATGCATCTTTTTTTCCT  2300
Consensuatctgtaattctgtagcctgacgatt               ccgaggtttctg                    gtgctaaaaaatgcatctttttttc t

C48-2    CAGATGACAATGCTTTCTGTCTTTGTTCACCGCAGATTCACAAGGGGTCTTCAGTGTTTGCGAGTGCAGTGGCCGGTAGGCTGATGGAAGTGGTGGACGA  2338
A619     CAGATGACAATGCTTTCTGTCTTTGTTCACCGCAGATTCACAAGGGGTCTTCAGTGTTTGCGAGTGCAGTGGCCGGTAGGCTGATGGAAGTGGTGGACGA  2400
Consensucagatgacaatgctttctgtctttgttcaccgcagattcacaaggggtcttcagtgtttgcgagtgcagtggccggtaggctgatggaagtggtggacga

C48-2    GTACTAGGCTACCATGCACTTGAATTTCTAGCTAGCTCTA..CGTACCGCGCTGCTATGAATCTAGCTATAGCGTTTCTTGGATGAAAGACT           2428
A619     GTACTAGGCTACCATGCACTTGAATTTCTAGCTAGCTCTACACGTACCGCGCTGCTATGAATCTAGCTATAGCGTTTCTTGGATGAAAGACT           2492
Consensugtactaggctaccatgcacttgaatttctagctagctcta  cgtaccgcgctgctatgaatctagctatagcgtttcttggatgaaagact
```
