# Supplementary material for: A preliminary identification of Rf*-A619, a novel restorer gene for CMS-C in maize (Zea mays L.)
Source: PeerJ. 2016 Nov 22;4:e2719. doi: 10.7717/peerj.2719 (PMC5126625; doi:10.7717/peerj.2719)

Figure S2 Confirmation of the specificity of primers F2/R2. (A) Comparison of the *Rf4* sequence in A619 and F2/R2 amplifying sequence. A619-Rf4 indicated the genome sequence of *Rf4* in the inbred line A619 and the number in it represent for bases position. (B) The search result of F2/R2 amplifying sequence in maize genome. The red bar reprsented for the position of F2/R2 amplifying sequence in maize B73 genome.

S2A

```
A619-Rf4                 (1152) ACCTGCTGCACTCGCTCGACGCCGCGGCGGCGGTCAATCCCGTCACGGCGCCGCCGCAGTCCACCATCGACTACTTCCTCGGCGGCGCCGATCCCCACCA        100
F2/R2 amplifying product        ACCTGCTGCACTCGCTCGACGCCGCGGCGGCGGTCAATCCCGTCACGGCGCCGCCGCAGTCCACCATCGACTACTTCCTCGGCGGCGCCGATCCCCACCA        100
Consensus                       acctgctgcactcgctcgacgccgcggcggcggtcaatcccgtcacggcgccgccgcagtccaccatcgactacttcctcggcggcgccgatccccacca

A619-Rf4                        GCAGGCCATGCAGTACGAGCCGCTGCCGCCCGCCGCGGGCGGCCACCACCAGTACACCATGGACATGTTCCGCGACTACTGCGACGGCAACTACCCCACC        200
F2/R2 amplifying product        GCAGGCCATGCAGTACGAGCCGCTGCCGCCCGCCGCGGGCGGCCACCACCAGTACACCATGGACATGTTCCGCGACTACTGCGACGGCAACTACCCCACC        200
Consensus                       gcaggccatgcagtacgagccgctgccgcccgccgcgggcggccaccaccagtacaccatggacatgttccgcgactactgcgacggcaactaccccacc

A619-Rf4                        GCCGAGCCGTACATCCGCGGGACAATGACTGGAGCCCTCGTGTTCGGGGCCACCGACGACGACGACTCGGCCGCTGCCTACATGCCCGGGGGGCACTTTG        300
F2/R2 amplifying product        GCCGAGCCGTACATCCGCGGGACAATGACTGGAGCCCTCGTGTTCGGGGCCACCGACGACGACGACTCGGCCGCTGCCTACATGCCCGGGGGGCACTTTG        300
Consensus                       gccgagccgtacatccgcgggacaatgactggagccctcgtgttcggggccaccgacgacgacgactcggccgctgcctacatgcccggggggcactttg

A619-Rf4                        AGACCTCCCCGCCGCCGCCACGCGCCACCGGCCGCGGCAGGAAGCGGGGCAGGGCGCTGGGCGGCGGCTTCCATGCTGTGCTGGCCAACGGCGTCGAGAA        400
F2/R2 amplifying product        AGACCTCCCCGCCGCCGCCACGCGCCACCGGCCGCGGCAGGAAGCGGGGCAGGGCGCTGGGCGGCGGCTTCCATGCTGTGCTGGCCAACGGCGTCGAGAA        400
Consensus                       agacctccccgccgccgccacgcgccaccggccgcggcaggaagcggggcagggcgctgggcggcggcttccatgctgtgctggccaacggcgtcgagaa

A619-Rf4                        GAAGGAGAAGCAGCGCCGGCTGCGGCTCACCGAGAAGTTTACGGCCCTCATGCACCTCATACCCAACGTTACGAAGGTCGTACGGCGTACTTGCGC (1647)   496
F2/R2 amplifying product        GAAGGAGAAGCAGCGCCGGCTGCGGCTCACCGAGAAGTTTACGGCCCTCATGCACCTCATACCCAACGTTACGAAGGTCGTACGGCGTACTTGCGC          496
Consensus                       gaaggagaagcagcgccggctgcggctcaccgagaagtttacggccctcatgcacctcatacccaacgttacgaaggtcgtacggcgtacttgcgc
```

S2B

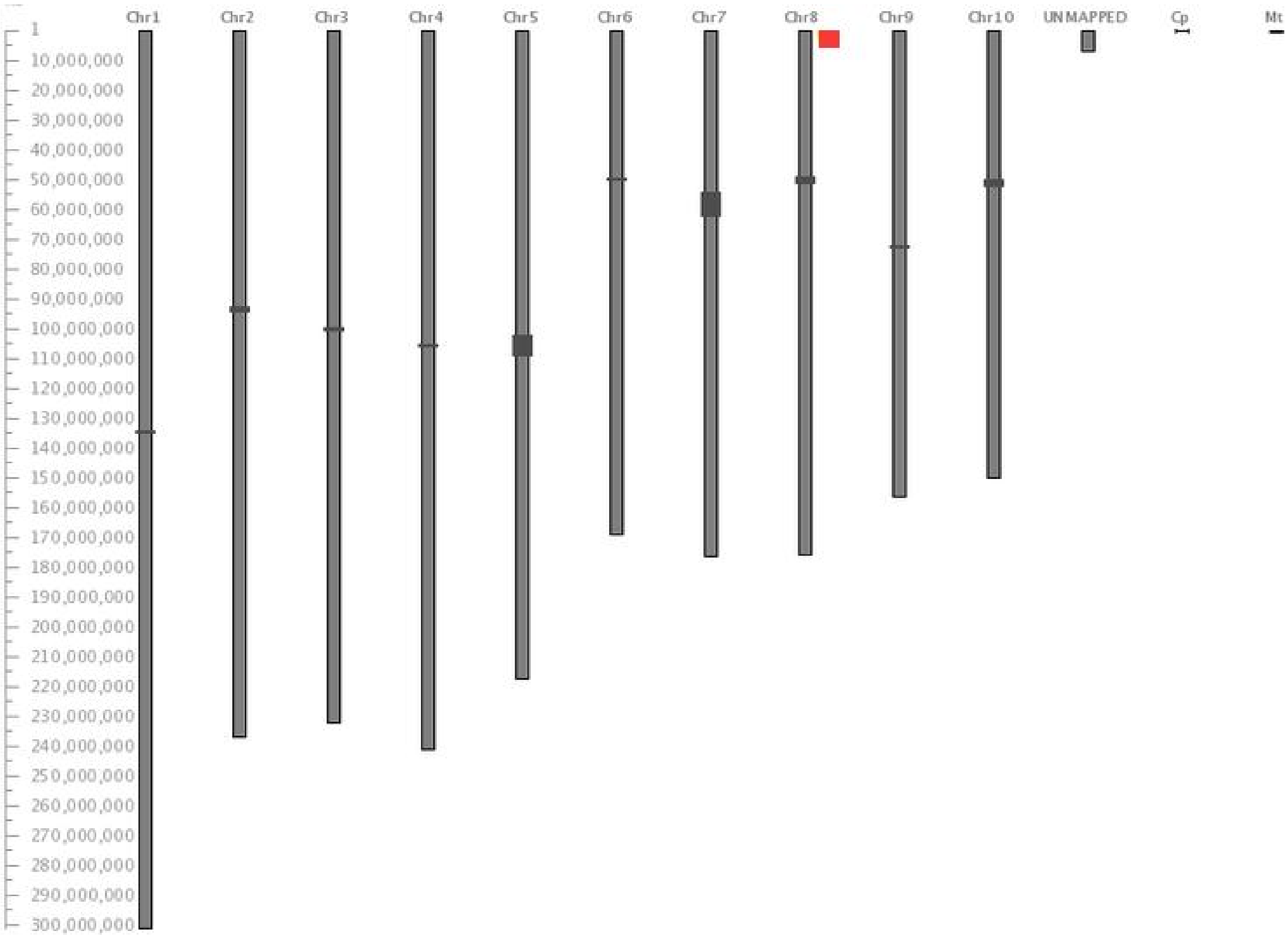

Supplement: Figure S2 — (A) Comparison of the Rf4 sequence in A619 and F2/R2 amplifying sequence. A619-Rf4 indicated the genome sequence of Rf4 in the inbred line A619 and the number in it represent for bases position. (B) The search result of F2/R2 amplifying sequence in maize genome. The red bar reprsented for the position of F2/R2 amplifying sequence in maize B73 genome. [file peerj-04-2719-s003.pdf]
